# Complete genome sequence of *Candidatus* Phytoplasma *australasiaticum* WF_GM2021, a plant pathogen associated with soybean witches’ broom disease in Taiwan

**DOI:** 10.1128/mra.00993-23

**Published:** 2024-01-11

**Authors:** Nian-Pu Li, Xiao-Hua Yan, Shen-Chian Pei, Ting-Hsuan Hung, Tsu-Cheng Chang, Qiong-Zhuan Bai, Chao-Feng Tai, Chih-Horng Kuo

**Affiliations:** 1 Institute of Plant and Microbial Biology, Academia Sinica, Taipei, Taiwan; 2 Department of Plant Pathology, National Chung Hsing University, Taichung, Taiwan; 3 Department of Plant Pathology and Microbiology, National Taiwan University, Taipei, Taiwan; 4 Agricultural Chemicals Research Institute, Ministry of Agriculture, Taichung, Taiwan; 5 Biotechnology Center, National Chung Hsing University, Taichung, Taiwan; The University of Arizona, Tucson, Arizona, USA

**Keywords:** genome, phytoplasma, pathogens, phytopathogens

## Abstract

The complete genome sequence of *Candidatus* Phytoplasma *australasiaticum* strain WF_GM2021, which consists of one 633,005-bp circular chromosome, is presented in this work. This uncultivated plant-pathogenic bacterium is associated with soybean (*Glycine max*) witches’ broom disease in Wufeng District, Taichung City, Taiwan.

## ANNOUNCEMENT

Phytoplasmas are plant-pathogenic bacteria that induce developmental abnormalities in their hosts, causing agricultural losses worldwide (1). Among the described *Candidatus* Phytoplasma species (2), *Ca*. P. *australasiaticum* is prevalent in Australia and Asia (3) and has been developed as a major representative for phytoplasma genomics research (4). To improve the sampling of these bacteria, a strain WF_GM2021 associated with soybean witches’ broom disease was collected for whole-genome sequencing. Unless stated otherwise, the methods were based on the cited references; the kits were used according to the manufacturer’s instructions; and the bioinformatics tools were used with the default settings.

A soybean plant with witches’ broom symptom was collected in Wufeng District (Taichung City, Taiwan; 24.01495 N 120.69428 E) in August 2021. The uncultivated phytoplasma was transmitted via dodder (*Cuscuta australis*) to periwinkle (*Catharanthus roseus*) for maintenance (5). After propagation via side grafting for one passage, the leaves of a periwinkle plant showing yellowing and witches’ broom symptoms were collected for DNA extraction without cultivation using a cetyltrimethylammonium bromide method (6). For Illumina sequencing, the DNA sample was processed using KAPA LTP Library Preparation Kit (Roche, Switzerland) and sequenced on a NovaSeq 6000 using S4 Reagent Kit v1.5 (Illumina, USA). A total of 87,826,828 pairs of 151-bp paired-end reads were generated. After quality trimming using Trimmomatic v.0.39 (7) with the “TrimmomaticPE” mode, 83,518,753 read pairs with a combined length of 24,467,418,087 bp were obtained.

By mapping reads to representative phytoplasma genomes that originated from Asian countries (4) using BWA v.0.7.17 (8), WF_GM2021 was found to share 100% 16S rRNA gene sequence identity with strain NCHU2014 (National Center for Biotechnology Information assembly accession number GCA_001307505.2, from a symptomatic *Echinacea purpurea* plant collected in Wufeng District in June 2014) (9, 10). Therefore, a resequencing strategy was used with NCHU2014 as the reference without re-assembly. The BWA mapping result was programmatically checked using SAMtools v.1.9 (11) to identify the polymorphisms. Polymorphic sites that have a QUAL score above 100 in the variant call format file produced by SAMtools were manually inspected using IGV v.2.11.1 (12) both before and after correcting the polymorphisms. The chromosomal contig was determined to be complete and circular based on the mapping of read pairs that span both ends of the contig. The procedure of gene prediction and annotation was based on that described in our previous work (5, 13). For gene prediction, RNAmmer v.1.2 (14), tRNAscan-SE v.1.3.1 (15), and Prodigal v.2.6.3 (16) were used. The annotation was based on the homologs in NCHU2014 (9, 10) as identified by OrthoMCL v.1.3 (17). Putative secreted proteins were identified using SignalP v.5.0 (14) and filtered by TMHMM v.2.0 (15).

The assembly produced a single 633,005-bp circular chromosomal contig with 24.5% G + C content. Among the quality-trimmed reads, 8,709,260 reads (5.2% of total) can be mapped to this uncultivated bacterium, providing 1,455.7-fold coverage. The same as the reference genome, which shares 99.96% average nucleotide identity (18), this new assembly has *dnaA* as the first gene. The annotation contains 6 rRNA genes, 27 tRNA genes, 507 protein-coding genes, and 12 pseudogenes.

## Data Availability

This genome project has been deposited in National Center for Biotechnology Information (NCBI) under the accession number PRJNA1008853. The raw reads have been deposited at the NCBI Sequence Read Archive under the accession number SRX21482278. The genome sequence has been deposited at GenBank under the accession number CP133702.
